# Inequity of healthcare utilization on mammography examination and Pap smear screening in Thailand: Analysis of a population-based household survey

**DOI:** 10.1371/journal.pone.0173656

**Published:** 2017-03-10

**Authors:** Sukanya Chongthawonsatid

**Affiliations:** Faculty of Social Sciences and Humanities, Mahidol University, Nakhon Pathom, Thailand; Iran University of Medical Sciences, ISLAMIC REPUBLIC OF IRAN

## Abstract

Healthcare in Thailand is not equally distributed, and not all people can equally access healthcare resources even if they are covered by health insurance. To examine factors associated with the utilization of mammography examination for breast cancer and Pap smear screening for cervical cancer, data from the national reproductive health survey conducted by the National Statistical Office of Thailand in 2009 was examined. The survey was carried out on 15,074,126 women aged 30–59 years. The results showed that the wealthier respondents had more mammograms than did the lower-income groups. The concentration index was 0.144. The data on Pap smears for cervical cancer also showed that the wealthier respondents were more likely to have had a Pap smear than their lower-income counterparts. The concentration index was 0.054. Determinants of mammography examination were education, followed by health welfare and wealth index, whereas the determinants of Pap smear screening were wealth index, followed by health welfare and education. The government should support greater education for women because education was associated with socioeconomic status and wealth. There should be an increase in the number of screening campaigns, mobile clinics, and low-cost mammograms and continued support for accessibility to mammograms, especially in rural areas and low-income communities.

## Introduction

The problems of the Thai health system are mainly derived from inequities in the distribution of the healthcare budget, with the budget tending to be distributed preferentially to wealthier regions. This is reflected in the unequal distribution of high-tech medical devices, and although the number of such devices has increased, their inequitable distribution to provincial areas remains unresolved [1]. This inequality impacts upon women’s access to healthcare screening programs such as mammography examination for breast cancer and Pap smear screening for cervical cancer. The National Cancer Institute in Thailand reported that the breast cancer was ranked first in terms of incidence (42.41%) while cancer of the cervix uteri was ranked second (12.15%) of all female cancers in 2014 [2]. Mammography examination among women aged 40 years and over reduce breast cancer mortality [3] while Pap smear screening every 3 years between the ages of 20–75 years reduced the incidence of cervical cancer from 215 per 10,000 to 107 per 10,000 [4]. Breast and cervical cancer can both be detected at an early stage and are amenable to treatments that can reduce morbidity and mortality [5]. Under the Thai national health policy, women aged 30 to 60 or in a specific risk group should undergo mammography examination and Pap smear screening every year. In the national health examination survey IV, Thailand reported that 42.5% of women aged 15 to 59 years and only 4.5% of women aged 45 to 59 years underwent Pap smears and mammographies, respectively [6]. A previous study in Thailand found that mammograms were reported by 5.9% of women [7]. Not all people can easily access health services even if they are covered by health insurance. For example, healthcare centers and community hospitals in non-municipal or rural areas do not provide mammograms, and people from those areas need to be referred to provincial or large hospitals. However, to date there has been no formal analysis of the inequity of mammography examination and Pap smear screening in Thailand. The present study explored the inequity (access to health) of mammography examinations and Pap smear screenings by using a “concentration index”, determinant, and analyzed the factors that influence healthcare utilization of mammography examinations for breast cancer and Pap smear screenings for cervical cancer.

## Materials and methods

### Study population group and design

The data for the study were from the Thai National Statistical Office (NSO) survey conducted in Thailand in 2009. The reproductive health surveys were samples with a stratified two-stage sampling procedure. There are 76 provinces in Thailand, each of which was defined as a block/strata. Each stratum was separated into two parts according to the structure of the local administration, for instance, municipal and non-municipal areas. The municipal and non-municipal areas were set as the primary and secondary sampling units, respectively, including individual households in each area. The reproductive health surveys were population-based surveys that were systematically carried out by skilled interviewers who polled 15,074,126 women aged 30–59 years. The available data included each woman’s demographic background, socioeconomic status, types and frequency of health services utilization, medical expenses, health insurance coverage, breast cancer examination, and cervical cancer screening. The independent variables included their age, household characteristics, wealth index, education, occupation, health insurance, and area of residence. The dependent variable was their healthcare utilization (i.e., whether healthcare was used). As policy, women aged 30–59 years old should be screened by both mammography examination and Pap smear at least once a year. The questionnaire therefore asked the following questions about breast cancer examination and cervical cancer screening: “During last year, did you ever have your breasts examined?” Those who responded positively were asked a follow up question “During last year, did you ever have your breasts examined by a mammogram?”. Questions regarding cervical cancer screening were: “Have you ever had a cervical cancer screening?” and in the event of a positive response two further questions were asked: (i) “When was your last screening” (in years up to 5 years ago) and (ii) “Where did you have your cervical cancer screening?” If the response to the first question was negative a follow up question of: “What were the main reasons for not having cervical cancer screening?” was asked. While mammography examination can only be accessed at provincial or large hospitals, Pap smear screening can be accessed at all hospitals and healthcare centers.

### Analysis

Inequities in access to mammograms and Pap smears were analyzed using a concentration index. Two key variables, health and living standards, were used to construct the concentration curve. The wealth index was constructed by principal component analysis (PCA) of variables including type of dwelling construction material, home ownership or rental, and household assets, for example a wooden or metal bed, microwave, electric kettle, refrigerator, television, video, washing machine, air conditioner, a water heater machine in the bathroom, computer, telephone, mobile phone, car, small truck, pick-up, van, small farm machinery, and motorcycle. Then, the data were stratified into five living standard groups (i.e., assets quintiles that represented consecutive 20% portions of the wealth index), and the mean value of the health variable was calculated. The groups were ranked from the poorest (the lowest quartile) to the richest (the highest quintile).

The “concentration index” is related to the concentration curve and is a measure of economic inequality for a given health variable. The concentration index is defined in relation to the concentration curve, which is graphed on a plot where the x axis is the cumulative percentage of the economic factor, ranked by living standard from the poorest to the richest, and the y-axis is the cumulative percentage of a health variable. The concentration index is defined as double the area between the concentration curve and the line of equality (the 45-degree line running from the bottom-left corner to the top-right). There is no relation to the economic factor of inequality; i.e., the concentration index is zero. The principle is that the index is negative if the curve lies above the line of equality, indicating a concentration of health services among the poor, and it is positive if the curve lies below the line of equality [8]. The Gini coefficient is widely used to measure inequality in the distribution of income, wealth, expenditure, etc [9]. The study used decomposition of the Gini coefficients as a determinant of accessibility of mammograms for breast cancer and Pap smear screening by using Stata version 12. Logistic regression was used to assess factors associated with mammograms and Pap smears. Univariate logistic regression with p < 0.05 were adjusted in multiple logistic regression and backward stepwise (p<0.05). Multiple logistic regression analyses were performed to identify the factors associated with mammograms for breast cancer and Pap smears for cervical cancer by using Stata version 12.

### Ethics statement

The data used in this study was derived from the national reproductive health survey conducted by the National Statistical Office of Thailand in 2009. The present study was approved by the ethical committee of the Faculty of Social Sciences and Humanities, Mahidol University, Institutional Review Board certificate number 2015/054.1310.

## Results

**Fig 1:**
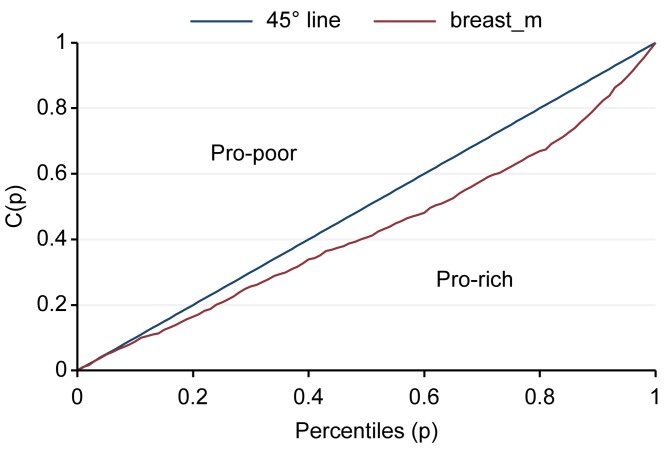
Concentration curve and concentration index of mammograms.

**Fig 2:**
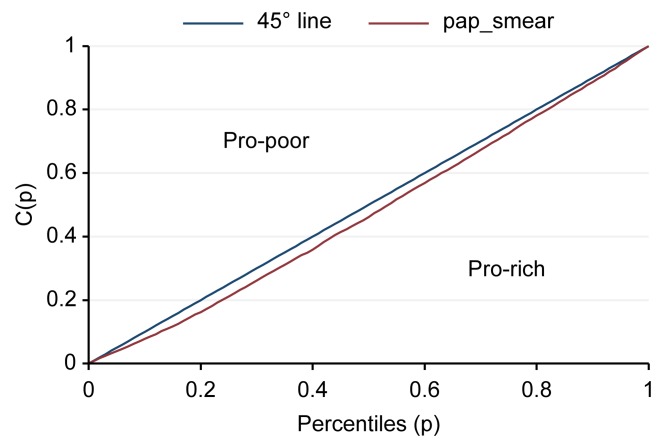
Concentration curve and concentration index of Pap smear screening.

The results of the coverage analysis of mammography examinations found that wealthier people had more mammograms than poorer people (pro-rich). The Concentration curve and concentration index of mammograms was 0.144 (95% CI = 0.089–0.199) (Fig 1). The results of coverage analysis of Pap smear screenings found that wealthier people had more Pap smears than poorer people (pro-rich). The Concentration curve and concentration index of Pap smear screening was 0.054 (95% CI = 0.031–0.077) (Fig 2). Determinants of mammography examination were education, followed by health welfare and wealth index, whereas those for Pap smear screening were wealth index, followed by health welfare and education. The occupation factor did not depend on mammography examination and Pap smear screening (R_k_ = -0.0932, -0.0416, respectively) (Table 1).

**Table 1:** Gini decomposition by income sources of mammography examination and Pap smear screening.

	Mammography examination					Pap smear				
Variables	S_k_	G_k_	R_k_	Share	% Change	S_k_	G_k_	R_k_	Share	% Change
Wealth index	5.7065	0.6369	0.1216	0.6406	-5.0659	2.0393	0.8472	0.1289	0.7157	-1.3236
Health welfare	8.0782	0.1606	0.1366	0.257	-7.8212	3.512	0.1514	0.0265	0.0453	-3.4667
Education	7.4821	0.2505	0.1668	0.4532	-7.029	3.2658	0.2481	0.0196	0.0511	-3.2147
Occupation	8.8491	0.2415	-0.0932	-0.2888	-9.1379	4.0728	0.2348	-0.0416	-0.1279	-4.2008
Total income		0.6896						0.3112		

S_k_ = share of source k in total income, G_k_ = the source Gini

R_k_ = Gini correlation of income from source *k* with the distribution of total income

Most of the respondents were aged 30–49 years, had an elementary level education (41.8%), worked in private enterprise and agriculture, farming, fisheries (70.6%), was Buddhist (94.7%), was married (78.3%), was aged between 20 and 29 years during their first marriage (56%), and lived in a rural area (66.5%). Only 2.7% of the sample did not have welfare. The fifth quintile of the wealth index comprised 32.5% of subjects, meaning that 32.5% of households had their own assets (such as a wooden or metal bed, microwave, electric kettle, refrigerator, television, video, washing machine, air conditioner, a water heater machine in the bathroom, computer, telephone, mobile phone, car, small truck, pick-up, van, small farm machinery, and motorcycle). The first and the second quintile were poor and rather poor households who did not own their own basic assets. The proportion of respondents who had had their breasts examined in the last year by a mammogram was only 29%, although the majority (58%) of the sample had self-examined their breasts and/or had been examined by health personnel. Under one-third (31.6%) had never had a Pap smear. The main reasons for not undergoing cervical cancer screening were lack of knowledge about the service and shyness. Most people were screened at a health center (Table 2).

**Table 2:** Characteristics of the sample.

Variables	Number	Percent
**Age (year)**		
30–39	5,561,306	36.9
40–49	5,487,316	36.4
50–59	4,025,504	26.7
Total	15,074,126	100.0
**Education**		
No education, kindergarten	4,011,781	26.6
Elementary	6,295,626	41.8
Secondary, vocational	3,243,685	21.5
Bachelor	1,334,349	8.9
Higher bachelor	188,685	1.2
Total	15,074,126	100.0
**Occupation**		
Government	991,713	6.6
Employee (private enterprise)	5,575,038	37.0
Agriculture, farming, fisheries	5,071,032	33.6
Laborer	1,323,651	8.8
No occupation	2,112,693	14.0
Total	15,074,126	100.0
**Religion**		
Buddhist	14,275,189	94.7
Muslim	722,106	4.8
Christian	59,645	0.4
Other (Hinduism, Confucius, Sikhism)	17,185	0.1
Total	15,074,126	100.0
**Marital status**		
Single	1,335,226	8.9
Married	11,799,852	78.3
Divorced	1,931,725	12.8
Not married	7,323	0.0
Total	15,074,126	100.0
**Age at first marriage or at the start of cohabitation**		
10–14	114,906	0.8
15–19	4,914,299	35.7
20–29	7,687,919	56.0
30–39	968,850	7.1
40–49	50,862	0.4
50–59	2,064	0.0
Total	13,738,900	100.0
**Residential area**		
Rural	10,021,052	66.5
Urban	5,053,074	33.5
Total	15,074,126	100.0
**Health welfare**		
No welfare	401,061	2.7
Universal coverage card	10,936,770	72.6
Social security/worker’s compensation fund	2,212,838	14.7
Civil servant medical benefits scheme	1,292,974	8.6
Other (private health insurance, health insurance covered by employer)	230,482	1.4
Total	15,074,126	100.0
**Wealth Index**		
Quintile: 1 –lowest 20%	1,324,037	8.8
Quintile: 2 –lower 20%	172,424	1.1
Quintile: 3 –middle 20%	4,263,905	28.3
Quintile: 4 –higher 20%	4,419,983	29.3
Quintile: 5 –highest 20%	4,893,777	32.5
Total	15,074,126	100.0
**Breast cancer examination**		
Ever had breasts examined		
Yes, self-exam	3,557,486	23.5
Yes, health personnel	3,055,759	20.3
Yes, self-exam and health personnel	2,144,716	14.2
No	6,316,165	42.0
Total	15,074,126	100.0
Ever had breast examined by a mammogram		
Yes	1,510,326	29.0
No	3,690,149	71.0
Total	5,200,475	100.0
**Cervical cancer screening (Pap smear)**		
Ever had cervical cancer screening (the latest one)		
Ever, 1 year	5,645,834	37.5
Ever, 2 years	2,003,715	13.3
Ever, 3 years	791,362	5.2
Ever, 4 years	351,485	2.3
Ever, 5 years	284,208	1.9
More than 5 years	1,227,809	8.2
Never	4,769,713	31.6
Total	15,074,126	100.0
Location of cervical cancer screening		
Health center/public health center	4,220,615	41.0
Public health center (in Bangkok)	42,694	0.4
Community hospital	1,558,714	15.1
Regional/general hospital	1,748,947	17.0
University hospital	192,882	1.9
Other public hospital	860,385	8.3
Private hospital/clinic	1,620,657	15.7
Mobile clinic	53,816	0.5
Other	5,703	0.1
Total	10,304,413	100.0
Main reason for not having a cervical cancer screening		
Did not know it’s needed	1,110,939	23.3
Felt shy	1,270,482	26.6
Afraid of pain	702,600	14.7
Difficult to access health center	181,064	3.8
Can’t afford the transportation cost	174,359	3.7
Healthy/no risk	746,731	15.7
No need for an exam/afraid of the results	137,898	2.9
No time	376,077	7.9
Other	69,564	1.4
Total	4,769,714	100.0

The univariate logistic regression results presented in Table 3 shows the relationships of demographic, socioeconomic, geographic, benefits scheme, and economic status factors with mammograms and Pap smear screening. Subjects with a higher education level were more likely to have had a mammogram than those with a lower education level. Subjects who worked for the government were more likely to have had a mammogram and Pap smear than those who were otherwise employed or were unemployed. Muslims, Christians, Hindus, Confucians, and Sikhs were less likely to have a Pap smear than Buddhists. Subjects who lived in urban areas were more likely to have had a mammogram than those who lived in rural areas, whereas rural areas were more likely to have had a Pap smear than those who lived in urban areas. Subjects in the highest strata of the wealth index were more likely to have had a mammogram and Pap smear than those in the lowest strata of the wealth index. Subjects who had civil servants medical benefits scheme were more likely to have had a mammogram than were those who did not have welfare or other groups. Subjects who had welfare were more likely to have had a Pap smear than were those who did not have welfare (Table 3).

**Table 3:** Relationships of demographic, socioeconomic, geographic, benefits scheme, and economic status factors with mammograms and Pap smears using Univariate Logistic Regression.

Variables	Mammography			Pap smear		
	OR	95%CI	p-value	OR	95%CI	p-value
***Demographic characteristics***						
Age	1.01	1.01–1.02	<0.001	1.01	1.01–1.02	<0.001
**Religion**						
Buddhist®	1		<0.001	1		<0.001
Muslim	1.07	1.06–1.08	<0.001	0.54	0.54–0.55	<0.001
Christian	1.00	0.97–1.03	0.998	0.69	0.68–0.71	<0.001
Other (Hinduism, Confucius, Sikhism)	1.60	1.48–1.74	<0.001	0.25	0.24–0.26	<0.001
***Geographic characteristics***						
Residential area						
Rural ®	1			1		
Urban	2.49	2.48–2.50	<0.001	0.66	0.65–0.66	<0.001
***Socioeconomic characteristics***						
Education						
No education, kindergarten ®	1		<0.001	1		<0.001
Elementary	1.23	1.22–1.24	<0.001	1.08	1.08–1.09	<0.001
Secondary, vocational	2.00	1.99–2.01	<0.001	0.99	0.99–1.00	0.067
Bachelor’s degree	4.07	4.05–4.10	<0.001	1.12	1.11–1.12	<0.001
Higher than bachelor’s degree	5.75	5.66–5.83	<0.001	0.99	0.98–1.00	0.245
Occupation						
Government ®	1		<0.001	1		<0.001
Private enterprise	0.55	0.54–0.55	<0.001	0.62	0.61–0.62	<0.001
Agriculture, farming, fisheries	0.26	0.25–0.26	<0.001	1.10	1.10–1.11	<0.001
Laborer	0.29	0.28–0.29	<0.001	0.52	0.52–0.53	<0.001
No occupation	0.55	0.55–0.56	<0.001	0.57	0.56–0.57	<0.001
***Economic status***						
Wealth index						
Quintile: 1 –lowest 20% ®	1		<0.001	1		<0.001
Quintile: 2 –lower 20%	0.66	0.64–0.68	<0.001	0.85	0.84–0.86	<0.001
Quintile: 3 –middle 20%	0.82	0.81–0.82	<0.001	1.55	1.54–1.55	<0.001
Quintile: 4 –higher 20%	0.76	0.75–0.76	<0.001	2.09	2.08–2.09	<0.001
Quintile: 5 –highest 20%	1.66	1.65–1.67	<0.001	2.49	2.48–2.50	<0.001
***Benefits scheme***						
Health welfare						
No welfare®	1		<0.001	1		<0.001
Universal coverage card	0.52	0.51–0.52	<0.001	3.17	3.15–3.19	<0.001
Social security/worker’s compensation fund	1.01	0.99–1.03	0.086	2.32	2.30–2.33	<0.001
Civil servants medical benefits scheme	1.50	1.47–1.52	<0.001	4.97	4.93–5.01	<0.001
Other (private health insurance, health insurance covered by employer)	1.08	1.05–1.09	<0.001	3.88	3.83–3.92	<0.001

® Reference group

Multiple regression analysis was performed to identify factors that were independently associated with mammograms and Pap smear screening. All variables with statistics significances in univariate analysis were entered into the logistic regression model using the backwards stepwise method. Results presented in Table 4 shows the relationships of demographic, socioeconomic, geographic, benefits scheme, and economic status factors with mammograms and Pap smear screening. Subjects with a higher education level were more likely to have had a mammogram and Pap smear than those with a lower education level. Subjects who worked in agriculture, farming and fisheries were more likely to have had a Pap smear than those who worked for the government. Muslims, Christians, Hindus, Confucians, and Sikhs were less likely to have a Pap smear and mammogram than Buddhists. Subjects who lived in urban areas were more likely to have had a mammogram than those who lived in rural areas, whereas rural areas were more likely to have had a Pap smear than those who lived in urban areas. Subjects in the highest strata of the wealth index were more likely to have had a mammogram and Pap smear than those in the lowest strata of the wealth index. Subjects who had welfare were more likely to have had a Pap smear than were those who did not have welfare, whereas subjects who had welfare were less likely to have had a mammogram examination than were those who did not have welfare (Table 4)

**Table 4:** Relationships of demographic, socioeconomic, geographic, benefits scheme, and economic status factors with mammograms and Pap smear using Multiple Logistic Regression, Backward stepwise.

Variables	Mammography			Pap smear		
	OR	95%CI	p-value	OR	95%CI	p-value
***Demographic characteristics***						
Age	1.02	1.02–1.02	<0.001	1.00	1.00–1.00	<0.001
**Religion**						
Buddhist®	1		<0.001	1		<0.001
Muslim	1.06	1.04–1.07	<0.001	0.57	0.56–0.57	<0.001
Christian	0.60	0.58–0.62	<0.001	0.88	0.87–0.89	<0.001
Other (Hinduism, Confucius, Sikhism)	0.49	0.46–0.54	<0.001	0.70	0.67–0.72	<0.001
***Socioeconomic characteristics***						
Education						
No education, kindergarten ®	1		<0.001	1		<0.001
Elementary	1.35	1.34–1.35	<0.001	1.16	1.15–1.16	<0.001
Secondary, vocational	1.81	1.80–1.82	<0.001	1.19	1.19–1.20	<0.001
Bachelor’s degree	2.61	2.59–2.64	<0.001	1.20	1.19–1.20	<0.001
Higher than bachelor’s degree	3.31	3.25–3.36	<0.001	1.00	0.99–1.01	0.985
Occupation						
Government ®	1		<0.001	1		<0.001
Private enterprise	1.02	1.01–1.03	<0.001	0.92	0.92–0.93	<0.001
Agriculture, farming, fisheries	0.78	0.77–0.79	<0.001	1.49	1.48–1.50	<0.001
Laborer	0.77	0.76–0.77	<0.001	0.85	0.84–0.86	<0.001
No occupation	1.07	1.06–1.08	<0.001	0.81	0.81–0.82	<0.001
***Economic status***						
Wealth index						
Quintile: 1 –lowest 20% ®	1		<0.001	1		<0.001
Quintile: 2 –lower 20%	0.98	0.95–1.01	0.155	0.91	0.90–0.91	<0.001
Quintile: 3 –middle 20%	1.22	1.21–1.23	<0.001	1.46	1.45–1.47	<0.001
Quintile: 4 –higher 20%	1.08	1.07–1.09	<0.001	1.89	1.88–1.89	<0.001
Quintile: 5 –highest 20%	1.40	1.39–1.41	<0.001	2.51	2.50–2.52	<0.001
***Geographic characteristics***						
Residential area						
Rural ®	1			1		
Urban	1.63	1.62–1.64	<0.001	0.73	0.72–0.73	<0.001
***Benefits scheme***						
Health welfare						
No welfare®	1		<0.001	1		<0.001
Universal coverage card	0.73	0.72–0.75	<0.001	2.33	2.32–2.35	<0.001
Social security/worker’s compensation fund	0.88	0.87–0.90	<0.001	2.14	2.13–2.16	<0.001
Civil servants medical benefits scheme	0.96	0.95–0.98	<0.001	3.51	3.48–3.54	<0.001
Other (private health insurance, health insurance covered by employer)	0.80	0.79–0.82	<0.001	3.04	3.01–3.08	<0.001

® Reference group

## Discussion

The result of the coverage analysis of mammograms was that richer people had more mammograms than poorer people (pro-rich). Similarly, the result of the coverage analysis of Pap smears was that richer people had more Pap smears than poorer people (pro-rich). These results show the inequity of access to healthcare between richer and poorer people for mammograms and Pap smears. Determinants of mammography examination were education, followed by health welfare, and wealth index factors, whereas Pap smear screening were wealth index, followed by health welfare, and education factors. Rich households often had more educated residents, enabling them to seek for good health and good services. In agreement with previous studies, financial barriers can prevent those in the poorest socioeconomic status group from utilizing the screening and treatment services for the early diagnosis and subsequent treatment of breast cancer [10–12]. The study showed that residential area was associated with mammogram examination and Pap smear screening. Subjects who lived in urban areas were more likely to have had a mammogram than those who lived in rural areas, whereas rural areas were more likely to have had a Pap smear than those who lived in urban areas. In Thailand, mammograms are available at provincial, university and large private hospitals because they have the requisite equipment and radiologists on staff. Thailand has a shortage of radiologists. People living in rural areas need to travel to a provincial or private hospital in an urban area to have a mammogram. While various health benefit programs provide coverage for mammograms, some rural clients may be unable or unwilling to pay for the cost of travel to a hospital in the provincial capital and many rural areas or communities only have occasional campaigns that promote mammograms. This result is similar to a previous study that found that the time to travel to the nearest mammography center was also predictive of not having a mammogram [13]. The distance to a clinic was significantly associated with screening such that increased distance increased the odds of having an abnormal mammogram [14]. This study found that factors associated with mammography examination and Pap smear were age, religion, education, occupation, residential area, wealth index, and health welfare.

For Pap smears, health centers or community hospitals can collect a specimen and send it to a reference hospital for analysis with the results being returned to the health center or community hospital. Thus, access to Pap smears should be universal in Thailand. Previous studies have found that the factors associated with breast and cervical cancer screening were being educated at the bachelor's level or higher, being in the richest wealth quintile based on household wealth index, and being covered by the civil servants medical benefit scheme [7, 15–16]. Income, education, family doctor, age, and health insurance were independent predictors of the low utilization rate of mammograms and breast self-exams [17]. However, other factors, such as ignorance about the screening or shyness, may prevent some women from having the test. The major limitation of this study is that it is based on national survey data that was carried out all areas in Thailand. As such the questions were pre-defined and sharply limited, without the possibility for further elaboration. There have unanswered questions. Wealth index showed economic status and was constructed by assets ownership (e.g. a wooden or metal bed, microwave, electric kettle, refrigerator, television, video, washing machine, air conditioner, a water heater machine in the bathroom, computer, telephone, mobile phone, car, small truck, pick-up, van, small farm machinery, and motorcycle.) because the data did not obtain details on income variables, and the data did not differentiate by the number of assets in the households. This study used the questions on breast examination examination and Pap smear screening to proxy variables of accessibility of healthcare, because all at risk women should access these services. Based on the findings of the present study, the government should support women’s education because education is associated with socioeconomic status and wealth. Moreover, health education helps people increase their knowledge about mammograms and Pap smears, promoting early detection and treatment. There should be an increase in the number of campaigns that promote cancer screenings, mobile mammograms, Pap smears, and low-cost mammograms, especially in rural areas and lower-income communities. Finally, there should be policies and plans to increase the number of essential health sector personnel, especially radiologists who can perform mammograms in community hospitals.

## Conclusion

Analysis of inequity of healthcare utilization on mammogram examination and Pap smear screening showed that richer people had more mammograms and Pap smear screening than poorer people. Determinants of mammography examination were education, followed by health welfare and wealth index, whereas Pap smear screening were wealth index, followed by health welfare and education. The government should support greater education for women because education was associated with socioeconomic status and wealth. There should be an increase in the number of screening campaigns, mobile clinics, and low-cost mammograms and continued support for accessibility to mammograms, especially in rural areas and low-income communities.
